# Supplementary material for: Slow Dissociation of Nitazenes from the μ-Opioid Receptor Underlies the Challenge of Overdose Reversal
Source: bioRxiv. 2026 Apr 16:2026.04.14.718203. Preprint. [Version 1] doi: 10.64898/2026.04.14.718203 (PMC13104949; doi:10.64898/2026.04.14.718203)
Supplement: Supplement 1 [file media-1.pdf]

Slow Dissociation of Nitazenes from the μ-Opioid Receptor Underlies the Challenge of Overdose Reversal

Joseph Clayton,[†,‡] Laura B. Kozell,[¶,§] Amy J. Eshleman,[¶,§] Shelley H. Bloom,[¶,§] William E. Schutzer,[¶,§] Atheir I. Abbas,[¶,§,‖] Lidiya Stavitskaya,[*,‡] and Jana Shen[*,†]

†Department of Pharmaceutical Sciences, University of Maryland School of Pharmacy, Baltimore, Maryland 21201, U.S.A.

‡Division of Applied Regulatory Science, Office of Clinical Pharmacology, Office of Translational Sciences, Center for Drug Evaluation and Research, United States Food and Drug Administration, Silver Spring, Maryland 20993, U.S.A.

¶VA Portland Health Care System, Portland, Oregon 97239, U.S.A.

§Department of Psychiatry, Oregon Health & Science University, Portland, Oregon 97239, U.S.A.

‖Department of Behavioral Neuroscience, Oregon Health & Science University, Portland, Oregon 97239, U.S.A.

E-mail: Lidiya.Stavitskaya@fda.hhs.gov; jana.shen@rx.umaryland.edu

Contents

Experimental Materials and Methods 3

Computational Methods and Protocols 5

Supplemental tables 9

Supplemental figures 10

List of Tables

S1 Measured half-lives of nitazenes and fentanyls at different naloxone concentrations 9

S2 Simulation estimated half-lives of nitazenes 9

List of Figures

S1 Saturation binding curves of radioligands 11

S2 Association curves of radioligands to the receptor 12

S3 Observed association rate vs. radioligand concentration 13

S4 Competitive displacement of a nitazene due to naloxone or nalmefene 14

S5 Inhibition of nitazenes binding due to naloxone or nalmefene 15

S6 Root-mean-square deviation of the ligand in conventional MD simulations 16

S7 Nitazene-subpocket 2 residue distances in conventional MD simulations 17

S8 Nitazene-subpocket 3 residue distances in conventional MD simulations 18

Experimental Materials and Methods

Chemicals. Protonitazene, etonitazene, and etodesnitazene were purchased from Cayman Chemical Company (Ann Arbor, MI). Other reagents were sourced from Millipore Sigma. The radiolabeling of nitazene compounds was performed by ViTrax, Inc. (Placentia, CA). Specific activities were 21–25, 43 and 38 Ci/mmol, for protonitazene, etonitazene, and etodesnitazene, respectively.

Cell culture. Rat C6 glioma cells were transfected with human μOR cDNA inserted into the plasmid pcDNA3.1neo (cDNA Resource Center, Bloomsburg, PA). Stably transfected cells (C6-hμOR) were selected with 700 µg/ml G418.

Membrane preparation. C6-hμOR cells were cultured in DMEM supplemented with 10% fetal bovine serum, penicillin/streptomycin, and 400 µg/ml G418. C6-hμOR cells were grown to confluency, plates were rinsed with calcium-magnesium free phosphate buffered saline (cmf-PBS), and cells were scraped from dishes into cmf-PBS and centrifuged at 1000 x g for 10 min at 4°C. The supernatant was removed. Membranes were washed by the addition of 50 mM Tris buffer (pH 7.4 at 4°C; Tris buffer), homogenized, and centrifuged at 27,000 x g for 20 min at 4°C. This step was repeated. Pellets were covered with 3 ml Tris buffer and frozen at -80°C for later use.

Saturation binding experiment. To determine the K_d and B_max values of radioligands at μOR, saturation binding experiments were conducted with the appropriate incubation times for equilibrium for each radioligand (Figure S1) using six concentrations with duplicate determinations of total and nonspecific binding (10 µM naloxone). The incubation was terminated by rapid filtration through Perkin Elmer Filtermat A filters presoaked in 0.05% polyethylenimine on a Tomtec cell harvester (Hamden, CT) using cold Tris buffer (50 mM, pH 7.4) with a 6-second wash. The filters were dried, spotted with scintillation cocktail, and counted for 2 min after a 4-h interval on a Perkin Elmer microbetaplate counter. Final radioligand concentrations ranged from 0.038–1.99 nM for [^{3}H]protonitazene, 0.003–1.72 nM for [^{3}H]etonitazene, and 0.144–2.80 nM for [^{3}H]etodesnitazene.

K_i measurement for naloxone and nalmefene. To determine the K_i of naloxone or nalmefene, competition curves with unlabeled naloxone (0.1 nM–10 µM, half log concentrations) were conducted with each [^{3}H]ligand at concentrations at or above the K_d value for each ligand with the

appropriate incubation time established for each radioligand. The incubation was terminated by rapid filtration as described above.

Measurement of association and dissociation kinetics. Association binding assays were performed in 96-well plates using 50 mM Tris buffer (pH 7.4 at 37°C). Nonspecific binding was determined with 10 µM naloxone. Cell membranes (45 to 60 µg protein) were incubated with the radioligand at 37°C at times ranging from 0.08 to 90 min, as appropriate for each radioligand, determined experimentally (Figure S2). The incubation was terminated by rapid filtration as described above.

Dissociation binding assays were performed as described above using the experimentally established equilibration time and the K_d for each radioligand. Triplicate or quadruplicate wells with buffer and cells were incubated at 37°C with [^{3}H]ligand followed by addition of naloxone at times ranging from 0.08 to 90 min, as appropriate for each radioligand, determined experimentally. The incubation was terminated by rapid filtration as described above.

Data analysis. GraphPad Prism (version 11.0 for Windows; GraphPad Software, Boston, Massachusetts USA, www.graphpad.com) was used for analyses of receptor saturation and binding assays, ligand-receptor association and dissociation assays.

For receptor saturation binding, data was analyzed for three or more independent assays. A non-linear fit one site analysis of specific bound [^{3}H]ligand (in fmol/mg protein) vs. free [^{3}H]ligand (in nM) was used to determine the equilibrium constant (K_d in nM) and maximal receptor binding sites (B_{max} in fmol/mg protein). The K_d was determined for each independent assay and used to calculate average values. An ordinary one-way ANOVA (p=0.0088, F=11.52) followed by Tukey's test for multiple comparisons was performed to determine whether nitazene K_d values were significantly different. The K_d for etodesnitazene was significantly higher than protonitazene (p = 0.038) or etonitazene (p = 0.008).

For receptor binding assays to determine K_i (nM) values, the data were normalized to specific binding in the absence of naloxone or nalmefene. Three or more independent assays were measured with duplicate determinations. Data from each independent assay was analyzed using a non-

linear sigmoidal dose-response (variable slope) with the 100-to-0 range to determine IC_{50} values and Hill coefficients. K_i values were calculated using the Cheng-Prusoff transformation: $K_i = IC_{50}/(1 + L/K_d)$, where L is the radioligand concentration and K_d is the binding affinity of the radioligand, as determined in the saturation binding experiment.

To determine the apparent or observed association rate k_{obs} (in min^{-1}), a non-linear one phase exponential analysis was performed on the measured [^{3}H] ligand counts per minute (cpm) from association binding assays (Figure S2). A total of 9 or 10 assays using different [^{3}H]ligand concentrations were generated for each radioligand. Data was collected at time points up to 60 minutes. Linear fits of k_{obs} versus radioligand concentration were used to calculate the association rate constant k_{on} (in $nM^{-1}min^{-1}$) for each radioligand (Figure S3).

Using dissociation assays, the dissociation rate of opioids was characterized by measuring [^{3}H]ligand cpm over time. Data was collected from three or more assays at time points up to 90 min. The half-life of dissociation ($t_{1/2}$) was determined by fitting this data to a one-phase exponential decay model.

Computational Methods and Protocols

System preparation. The structures of μOR bound to protonitazene, etonitazene, and etodesnitazene were taken from our previous modeling work.[1] Briefly, using three docking programs (Glide,[2] MOE[3] and AutoDock[4]), a series of nitro-containing and nitro-less nitazenes (including the three studied here) were docked to an active conformation of μOR using the co-crystal structure of μOR:BU72 complex (PDB 5C1M)[5] or the cryo-EM structure of μOR:mitragynine pseudoindoxyl complex (PDB 7T2G[6]) as a template. The docking resulted in three types of binding poses, based on whether the nitazene's benzimidazole is placed in the receptor's subpocket 1 (SP1, between transmembrane helices TM2 and TM3), subpocket 2 (SP2, between TM1, TM2, and TM7), or subpocket 3 (SP3, between TM5 and TM6). The stabilities of these poses were then analyzed using binding-pose metadynamics, conventional MD (cMD), and metadynamics-based free energy

simulations, which led to the conclusion that the SP2 binding pose is most stable.

The simulation system was prepared using CHARMM-GUI.[7] The μOR-nitazene complex structure was oriented in a bilayer of POPC lipids (roughly 175 lipids per leaflet) using PPM 2.0 web server.[8] Water molecules were added with a layer thickness of 22.5 Å. 78 sodium and 92 chloride ions were added to neutralize the system at pH 7.4 (assuming default protonation states for acidic and basic residues) and achieve an ionic strength of 150 mM. All histidines were set to the HID tautomer, including H297$^{6.52}$ in the orthosteric binding site which was shown to affect fentanyl's residence time at the receptor.[9] The protein and lipids were represented by the CHARMM36m protein[10] and CHARMM36 lipid force fields,[11] respectively, while water was represented by the CHARMM modified TIP3P model.[12,13] The force field parameters of sodium and chloride ions were taken from Refs[14,15] with the atom-pair specific adjustment (also known as NBFIX) to reduce overbinding of sodium to carboxylates.[16] The CHARMM modified TIP3P model was used to represent water.[12,13] The force field parameters of nitazenes were assigned using CHARMM-GUI,[7] which utilizes the CGenFF algorithm for assigning force field parameters based on analogy to compounds with optimized parameters.[17,18]

Our previous work followed the default preparation protocol of CHARMM-GUI,[7] which involved minimizing over 5,000 steps, followed by heating under constant volume to 300 K over 125 ps with restraints on the protein positions, lipid positions, and lipid dihedral angles. Restraints were gradually reduced over 2.25 ns under constant pressure, then 100 ns was conducted to refine the docked position of the nitazene. In this work, we further refined the docked position by conducting an additional 200 ns for μOR in complex with protonitazene, etonitazene, or etodesnitazene. Analysis of trajectories was conducted using CPPTRAJ.[19]

Metadynamics simulations. Following 300-ns MD simulation, 15 independent well-tempered metadynamics[20] simulations were conducted. Following our previous work,[21] two collective variables (CV) were used: the z-position of the center of mass (COM) of the nitazene relative to that of the orthosteric binding pocket, and the coordination number (CN). The COM of the binding pocket was defined as using the C_α atoms of residues Y75, Q124, N127, W133, L144, D147, Y148,

M151, F152, L232, K233, V236, A240, W293, I296, H297, V300, W318, H319, I322, and Y326. The CN represents the number of contacts between the nitazene and μOR, defined as

$$CN = \sum_{i\in\mu\text{OR}} \sum_{j\in\text{nitazene}} \frac{1-(d_{ij}/d_{\text{cut}})^8}{1-(d_{ij}/d_{\text{cut}})^{16}},$$

where d_{ij} is the distance between the heavy atoms i in μOR and j in the nitazene, and d_{cut} is the cutoff distance (set to 4.5 Å). Following our previous dissociation simulations,[21] each simulation was conducted until an unbound state was reached, defined as the z-position CV reaching 15 Å. The Gaussian weight parameter was set to 0.5 kcal/mol and the width was set to 0.5 Å for the z-position CV and 5 for the coordination CV. Hills were deposited every 20 ps, while the tempering parameter for well-tempered metadynamics was set to 14.

OpenMM v8.0[22] was used to run both the preparation protocol and the well-tempered metadynamics simulations; PLUMED v2.7[23] was used to control the metadynamics, via the OpenMM PLUMED plugin. Pressure was maintained at 1 bar using the Monte-Carlo barostat[24] with a coupling frequency of 100 steps, and with the x and y dimensions coupled isotropically. Temperature was controled by the Langavin thermostat[25] with a friction coefficient of 1. SETTLE[26] was used for water molecules, while SHAKE[27] was used for all other atoms.

Estimation of residence times at the receptor. Infrequent metadynamics[28,29] was used to estimate the nitazene-μOR residence time. For each metadynamics simulation, the real exit time t_{exit} was estimated by multiplying the simulation time (accelerated by metadynamics) by the running temporal average of the metadynamics potential, called the acceleration factor α[28]

$$\alpha =< \exp(\beta V_{meta}(t)) >$$

where $V_{meta}(t)$ is the bias potential at simulation time t. Following previous work[21,30] the t_{exit} of each fifteen metadynamics simulations was then used to form an empirical cumulative distribution function (CDF) and fit this to a theoretical CDF for a Poisson process, representing the probability of observing at least one dissociation event by time t:

$$P_{n\geq 1}(t) = 1 - \exp(-t/\tau)$$

where τ is the residence time. To estimate the errors of τ we performed bootstrapping analysis where the fifteen t_{exit} values were resampled 10,000 times. Each resampling was used to fit a Poisson process, and the Kolmogorov-Smirnov (KS) test was used to test if the sample and fitted Poisson process share the same underlying distribution.[30] If the sample showed a p-value lower than 0.05, then the sample was discarded. Remaining samples (where the p-value was equal or greater than 0.05) were used to calculate the mean and standard error of τ.

To produce unbiased probability distributions and consequentially the potential of mean force, frames were reweighted through an in-house script that follows the REWEIGHT_BIAS module implemented in PLUMED.[23]

Supplemental tables

Table S1: Experimentally determined $t_{1/2}$ (min) of nitazenes and fentanyls at different concentrations of unlabeled naloxone

Naloxone	10 nM	100 nM	1 μM	10 μM
Protonitazene	34.46±2.68	25.87±1.12	27.89±3.38	23.52±1.90
Etonitazene	14.15±0.64	10.87±0.14	10.30±0.10	
Etodesnitazene	8.25±1.13	6.34±0.18	5.37±0.50	
Fentanyl	4.27±0.53			3.67±0.48
Carfentanil	22.9±1.8			19.72±1.64

Three replicate experiments were conducted for each compound and naloxone concentration. Average and standard error are shown. For fentanyl and carfentanil, two naloxone concentrations were used in experiments.

Table S2: Simulation estimated $t_{1/2}$ for nitazenes. Each receptor-ligand system was subject to 15 independent metadynamics simulations, yielding 15 estimates of the unbiased dissociation times. The distribution was then fit to a theoretical Poisson process to obtain $t_{1/2}$. For estimation of statistical uncertainty, 10,000 bootstrapping trials were performed, keeping only trials that yielded a p-value above 0.15 (valid trials).

Compound	$t_{1/2}$ (s)	# Valid trials	Avg. p value
Protonitazene	18.3±0.3	6293	0.15
Etonitazene	0.26±0.02	7042	0.15
Etodesnitazene	0.0144±0.0006	4472	0.11
Fentanyl	1.469±0.017	6972	0.18

Supplemental figures

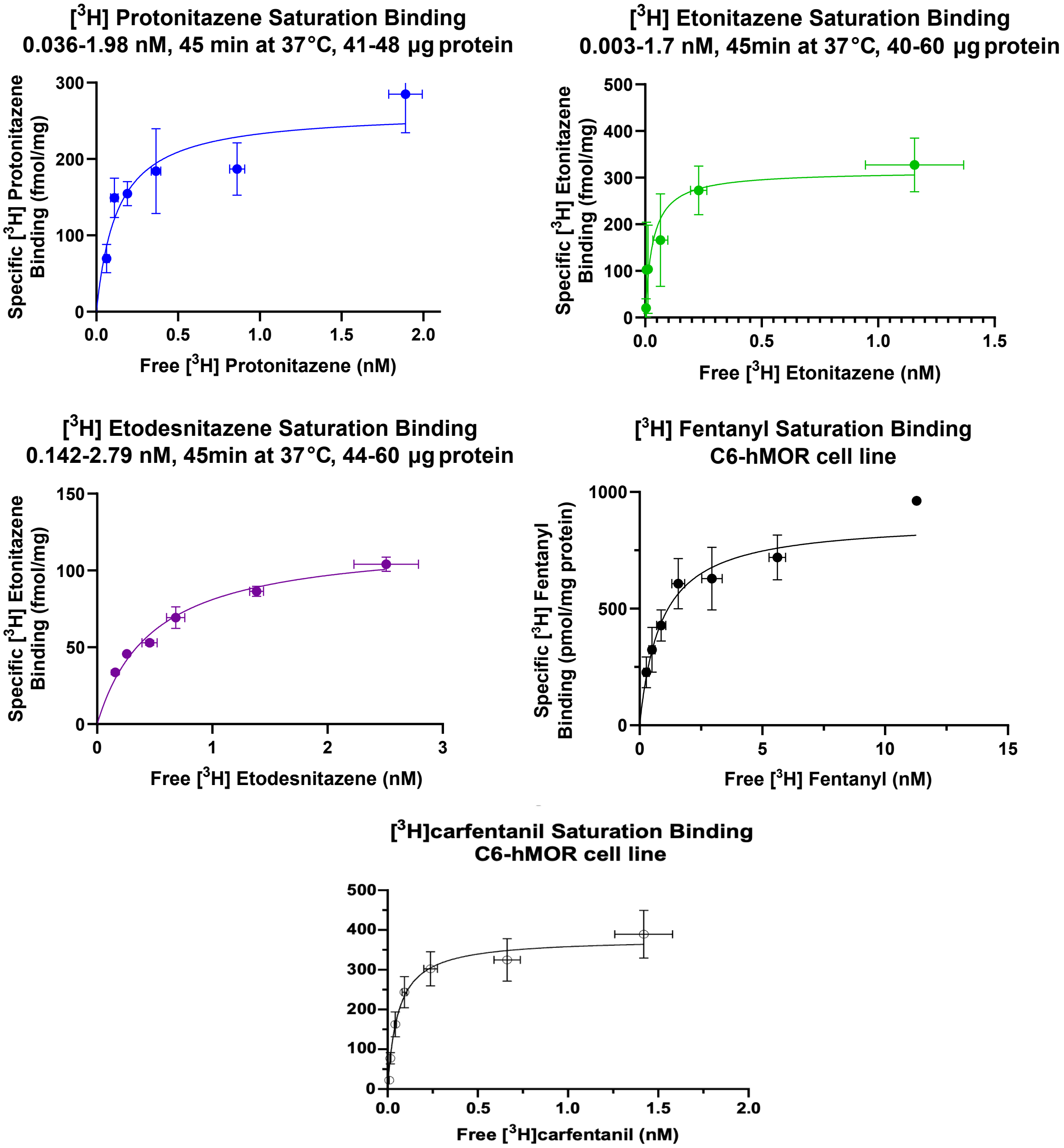

Figure S1: **Composite saturation binding curves for [^{3}H] nitazenes and [^{3}H] fentanyl binding to μOR**. Composite curves were generated using average specific bound [^{3}H]ligand vs. average free [^{3}H]ligand (nM) from 3 or 5 independent assays. Curves are the best fits to the one site specific binding model. Bars represent the standard error of the mean.

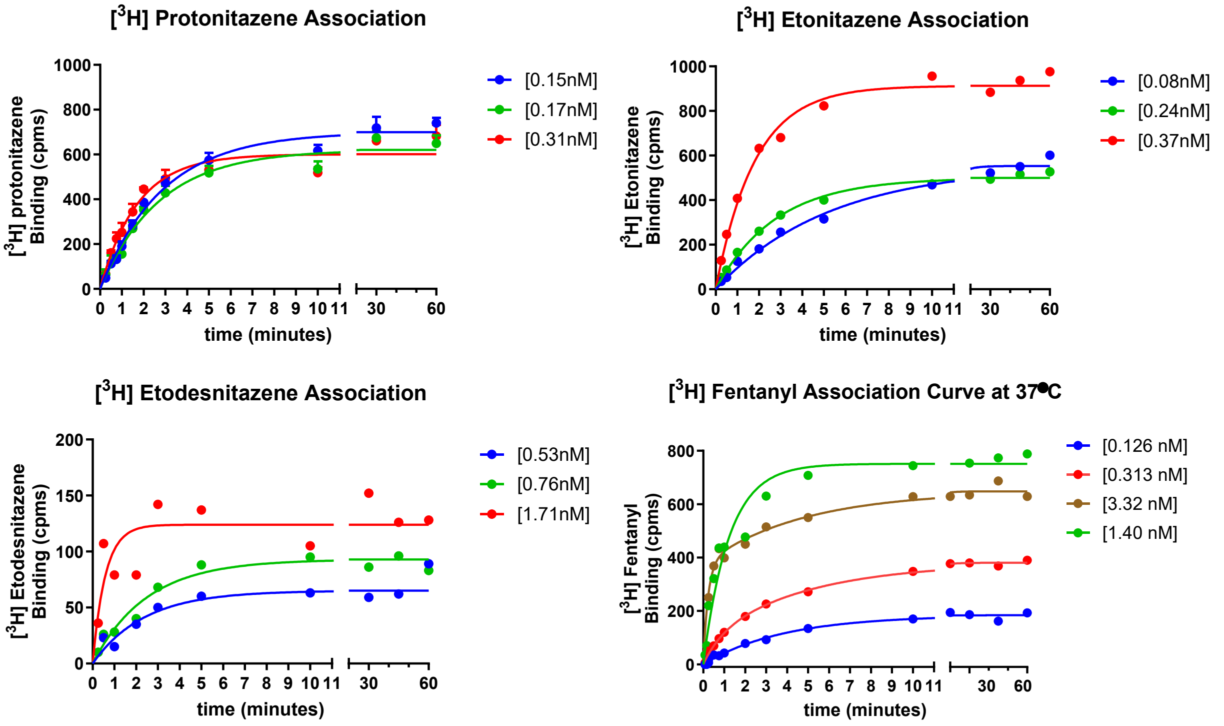

Figure S2: **Representative association curves showing the time course for association of [^{3}H]radioligands to the μOR**. One phase exponential (pseudo-first order) association curves corresponding to each association assay are shown. A total of 3 or 4 curves using different [^{3}H] radioligand concentrations are shown.

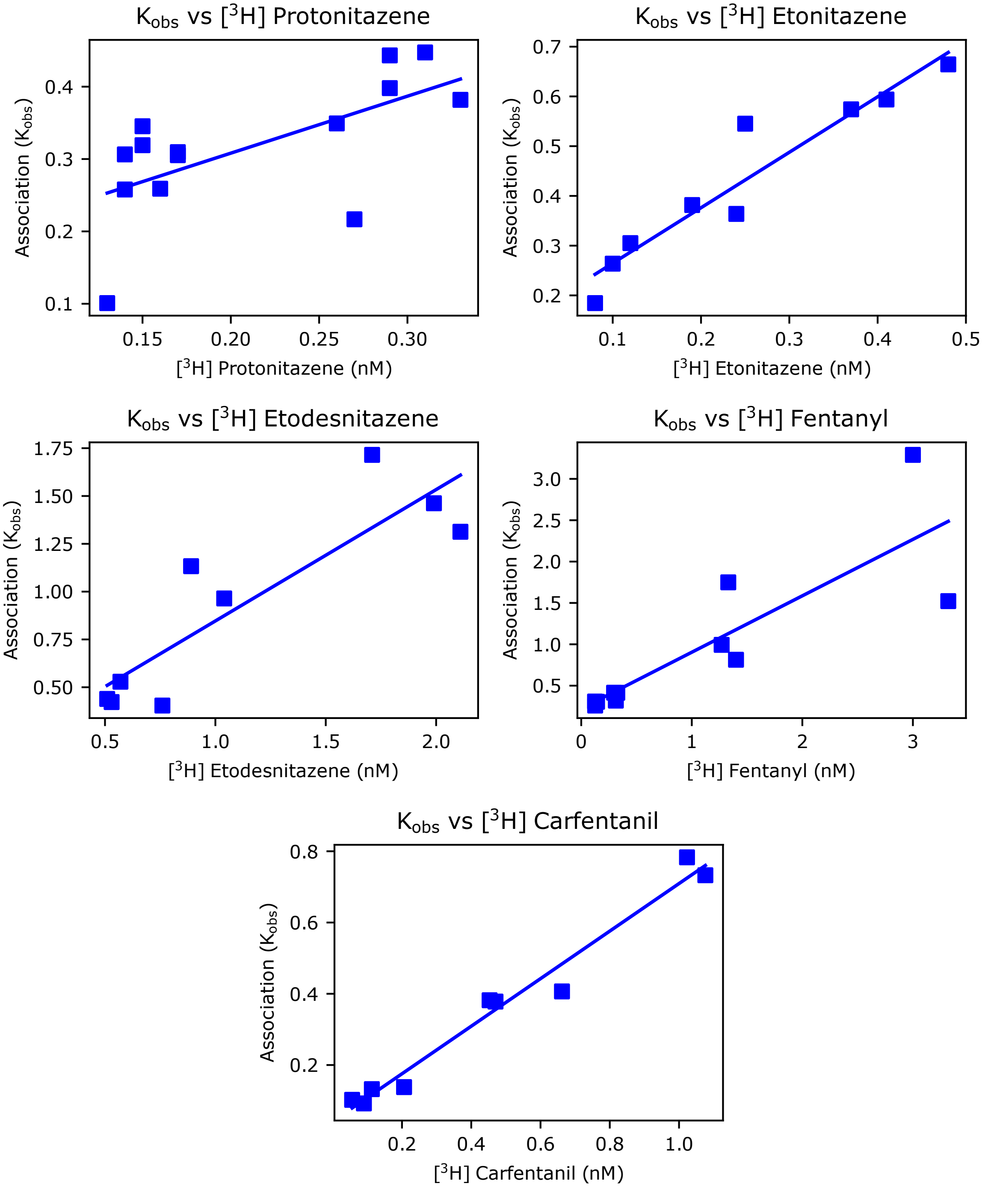

Figure S3: **Apparent association rate for [^{3}H]nitazenes and [^{3}H]fentanyl**. For each association assay, a one phase exponential association curve (see Figure S2) was used to calculate the apparent or observed association kinetic rate (k_{obs} in min^{-1}) at each radioligand concentration. A total of 9 or 13 concentrations were used. The panel above shows k_{obs} plotted versus the concentration of unbound radioligand (nM). Linear regression of the data yields the estimated k_{on} in nM^{-1}min^{-1} (slope of the line) for each radioligand.

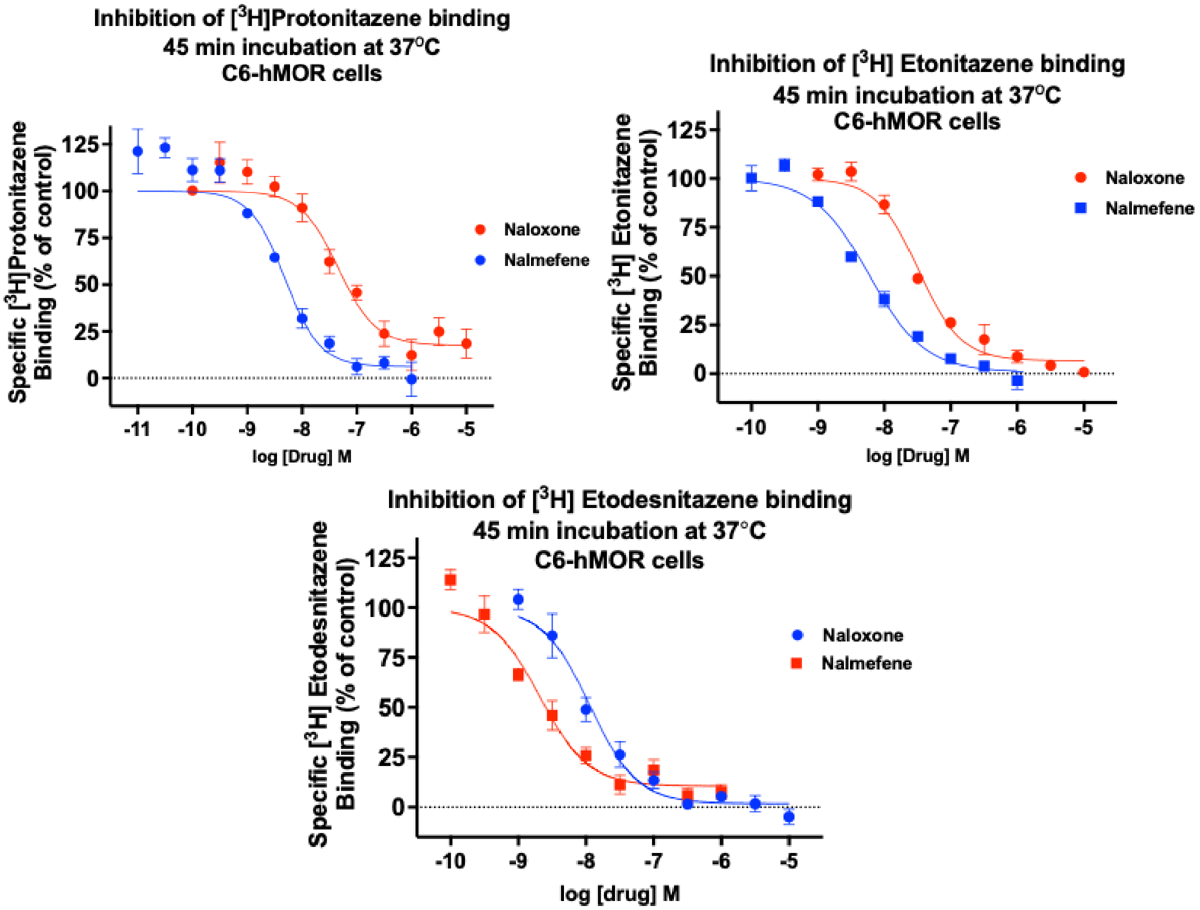

Figure S4: Competitive displacement of a nitazene due to naloxone or nalmefene at the μOR.

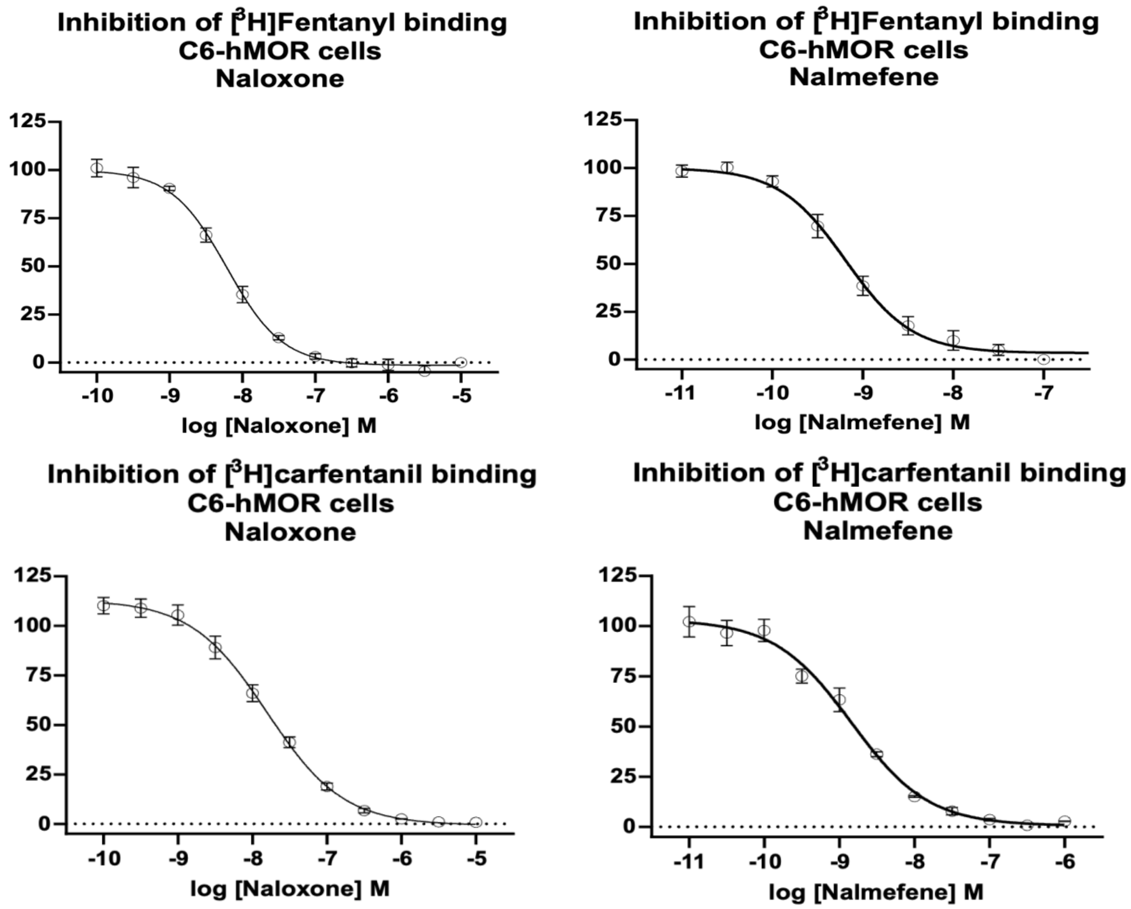

Figure S5: Competitive displacement of either fentanyl or carfentanyl due to naloxone or nalmefene at the μOR.

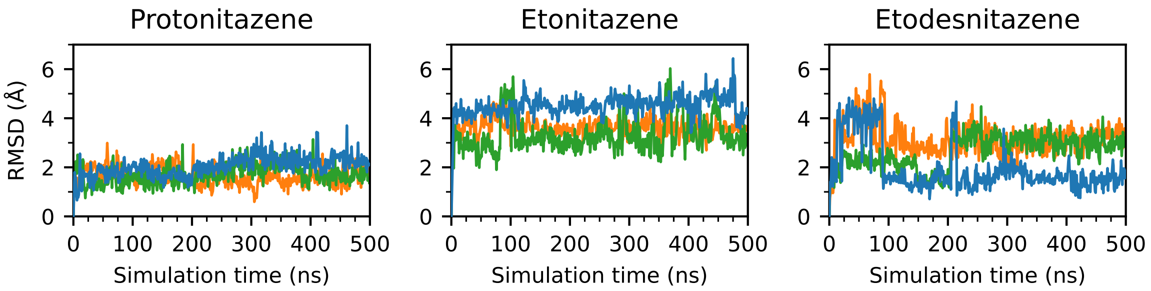

Figure S6: Root mean squared deviation (RMSD) of heavy atoms of the ligand during the three 500 ns conventional MD simulation, with respect to the docked structure. The starting conformation for estimating the dissociation $t_{1/2}$ was taken at 300 ns of the simulation show in blue.

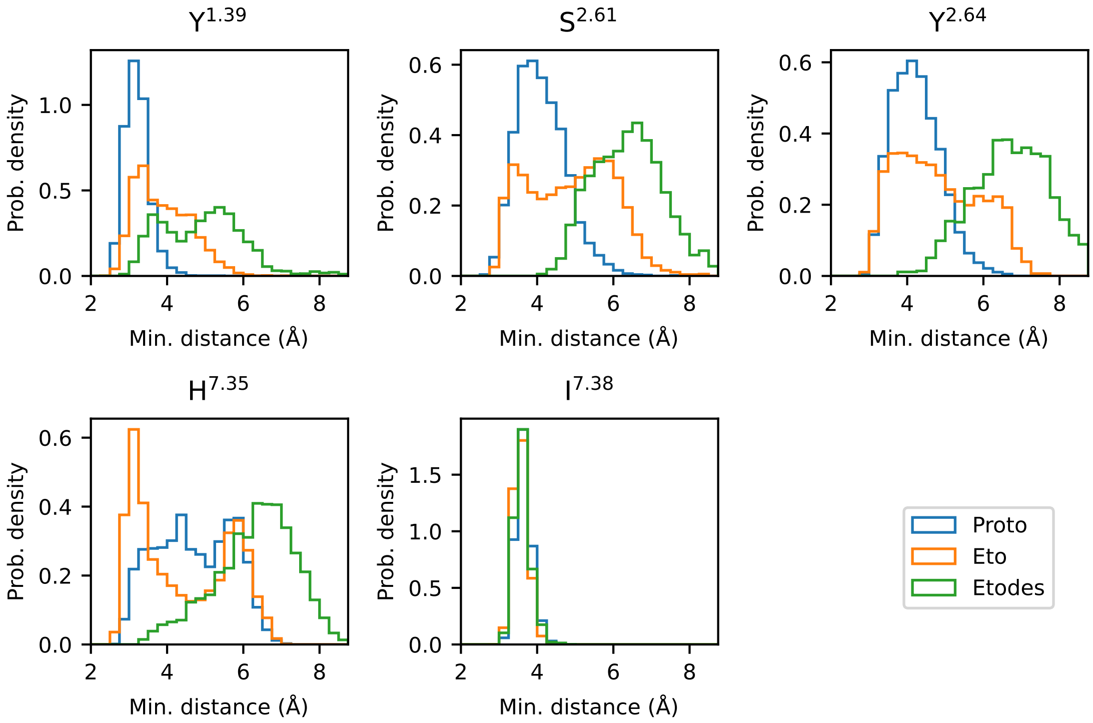

Figure S7: Minimum distance between non-hydrogen atoms of each nitazene and residues within SP2. Only the final 100 ns of three 300 ns simulations were analyzed.

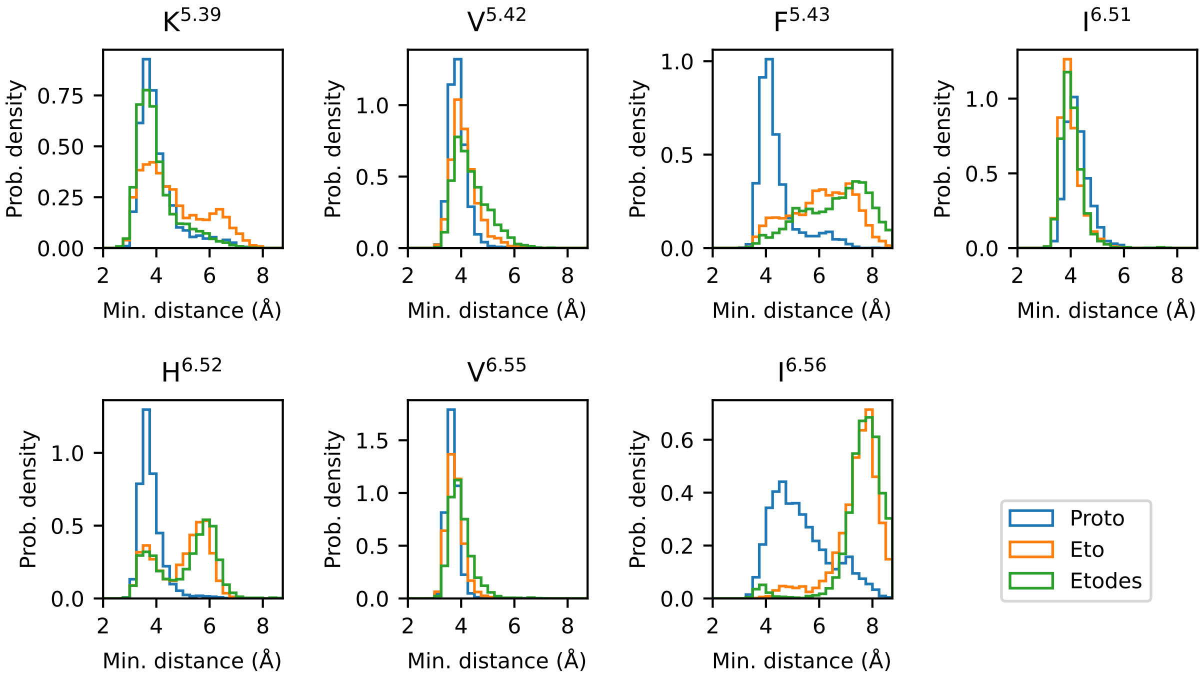

Figure S8: Minimum distance between non-hydrogen atoms of each nitazene and residues within SP3. Only the final 100 ns of three 300 ns simulations were analyzed.

References

(1) Clayton, J.; Shi, L.; Robertson, M. J.; Skiniotis, G.; Michaelides, M.; Stavitskaya, L.; Shen, J. A Putative Binding Model of Nitazene Derivatives at the μ-Opioid Receptor. *Neuropharmacology* **2025**, *273*, 110437.

(2) Friesner, R. A.; Banks, J. L.; Murphy, R. B.; Halgren, T. A.; Klicic, J. J.; Mainz, D. T.; Repasky, M. P.; Knoll, E. H.; Shelley, M.; Perry, J. K.; Shaw, D. E.; Francis, P.; Shenkin, P. S. Glide: A New Approach for Rapid, Accurate Docking and Scoring. 1. Method and Assessment of Docking Accuracy. *J. Med. Chem.* **2004**, *47*, 1739–1749.

(3) Molecular Operating Environment (MOE), 2022.02 Chemical Computing Group ULC. 910-1010 Sherbrooke St. W., Montreal, QC H3A 2R7, Canada.

(4) Eberhardt, J.; Santos-Martins, D.; Tillack, A. F.; Forli, S. AutoDock Vina 1.2.0: New Docking Methods, Expanded Force Field, and Python Bindings. *J. Chem. Inf. Model.* **2021**, *61*, 3891–3898.

(5) Huang, W. et al. Structural Insights into μ-Opioid Receptor Activation. *Nature* **2015**, *524*, 315–321.

(6) Qu, Q. et al. Insights into Distinct Signaling Profiles of the μOR Activated by Diverse Agonists. *Nat. Chem. Biol.* **2023**, *19*, 423–430.

(7) Jo, S.; Kim, T.; Iyer, V. G.; Im, W. CHARMM-GUI: A Web-Based Graphical User Interface for CHARMM. *J. Comput. Chem.* **2008**, *29*, 1859–1865.

(8) Lomize, M. A.; Pogozheva, I. D.; Joo, H.; Mosberg, H. I.; Lomize, A. L. OPM Database and PPM Web Server: Resources for Positioning of Proteins in Membranes. *Nucl. Acids. Res.* **2012**, *40*, D370–D376.

(9) Mahinthichaichan, P.; Vo, Q. N.; Ellis, C. R.; Shen, J. Kinetics and Mechanism of Fentanyl Dissociation from the μ-Opioid Receptor. *JACS Au* **2021**, *1*, 2208–2215.

(10) Huang, J.; Rauscher, S.; Nawrocki, G.; Ran, T.; Feig, M.; De Groot, B. L.; Grubmüller, H.; MacKerell, A. D. CHARMM36m: An Improved Force Field for Folded and Intrinsically Disordered Proteins. *Nat Methods* **2017**, *14*, 71–73.

(11) Klauda, J. B.; Venable, R. M.; Freites, J. A.; O'Connor, J. W.; Tobias, D. J.; Mondragon-Ramirez, C.; Vorobyov, I.; MacKerell, A. D.; Pastor, R. W. Update of the CHARMM All-Atom Additive Force Field for Lipids: Validation on Six Lipid Types. *J. Phys. Chem. B* **2010**, *114*, 7830–7843.

(12) Jorgensen, W. L.; Chandrasekhar, J.; Madura, J. D. Comparison of Simple Potential Functions for Simulating Liquid Water. *J. Chem. Phys.* **1983**, *79*, 926.

(13) MacKerell Jr., A. D. et al. All-Atom Empirical Potential for Molecular Modeling and Dynamics Studies of Proteins. *J. Phys. Chem. B* **1998**, *102*, 3586–3616.

(14) Beglov, D.; Roux, B. Finite Representation of an Infinite Bulk System: Solvent Boundary Potential for Computer Simulations. *The Journal of Chemical Physics* **1994**, *100*, 9050–9063.

(15) Lev, B.; Roux, B.; Noskov, S. Y. Relative Free Energies for Hydration of Monovalent Ions from QM and QM/MM Simulations. *J. Chem. Theory Comput.* **2013**, *9*, 4165–4175.

(16) Venable, R. M.; Luo, Y.; Gawrisch, K.; Roux, B.; Pastor, R. W. Simulations of Anionic Lipid Membranes: Development of Interaction-Specific Ion Parameters and Validation Using NMR Data. *J. Phys. Chem. B* **2013**, *117*, 10183–10192.

(17) Vanommeslaeghe, K.; MacKerell, A. D. Automation of the CHARMM General Force Field (CGenFF) I: Bond Perception and Atom Typing. *Journal of Chemical Information and Modeling* **2012**, *52*, 3144–3154.

(18) Vanommeslaeghe, K.; Raman, E. P.; MacKerell, A. D. Automation of the CHARMM General Force Field (CGenFF) II: Assignment of Bonded Parameters and Partial Atomic Charges. *Journal of Chemical Information and Modeling* **2012**, *52*, 3155–3168.

(19) Roe, D. R.; Cheatham, T. E. PTRAJ and CPPTRAJ: Software for Processing and Analysis of Molecular Dynamics Trajectory Data. *J. Chem. Theory Comput.* **2013**, *9*, 3084–3095.

(20) Barducci, A.; Bussi, G.; Parrinello, M. Well-Tempered Metadynamics: A Smoothly Converging and Tunable Free-Energy Method. *Phys. Rev. Lett.* **2008**, *100*, 020603.

(21) Mahinthichaichan, P.; Liu, R.; Vo, Q. N.; Ellis, C. R.; Stavitskaya, L.; Shen, J. Structure–Kinetics Relationships of Opioids from Metadynamics and Machine Learning Analysis. *J. Chem. Inf. Model.* **2023**, *63*, 2196–2206.

(22) Eastman, P. et al. OpenMM 8: Molecular Dynamics Simulation with Machine Learning Potentials. *J. Phys. Chem. B* **2024**, *128*, 109–116.

(23) Tribello, G. A.; Bonomi, M.; Branduardi, D.; Camilloni, C.; Bussi, G. PLUMED 2: New Feathers for an Old Bird. *Comput. Physics Commun.* **2014**, *185*, 604–613.

(24) Åqvist, J.; Wennerström, P.; Nervall, M.; Bjelic, S.; Brandsdal, B. O. Molecular Dynamics Simulations of Water and Biomolecules with a Monte Carlo Constant Pressure Algorithm. *Chem. Phys. Lett.* **2004**, *384*, 288–294.

(25) Loncharich, R. J.; Brooks, B. R.; Pastor, R. W. Langevin Dynamics of Peptides: The Frictional Dependence of Isomerization Rates of *N* -acetylalanyl- *N* ′-methylamide. *Biopolymers* **1992**, *32*, 523–535.

(26) Miyamoto, S.; Kollman, P. A. Settle: An Analytical Version of the SHAKE and RATTLE Algorithm for Rigid Water Models. *J. Comput. Chem.* **1992**, *13*, 952–962.

(27) Ryckaert, J.-P.; Ciccotti, G.; Berendsen, H. J. Numerical Integration of the Cartesian Equations of Motion of a System with Constraints: Molecular Dynamics of n-Alkanes. *J. Comput. Phys.* **1977**, *23*, 327–341.

(28) Tiwary, P.; Parrinello, M. From Metadynamics to Dynamics. *Phys. Rev. Lett.* **2013**, *111*, 230602.

(29) Valsson, O.; Tiwary, P.; Parrinello, M. Enhancing Important Fluctuations: Rare Events and Metadynamics from a Conceptual Viewpoint. *Annu. Rev. Phys. Chem.* **2016**, *67*, 159–184.

(30) Salvalaglio, M.; Tiwary, P.; Parrinello, M. Assessing the Reliability of the Dynamics Reconstructed from Metadynamics. *J. Chem. Theory Comput.* **2014**, *10*, 1420–1425.
